# Burden of typhoid fever in primary and hospital care in Indonesia: a serial cross-sectional study using national health insurance (BPJS Kesehatan) data, 2015–2023

**DOI:** 10.1016/j.lanwpc.2026.101994

**Published:** 2026-10-01

**Authors:** Vicka Oktaria, Achmad Dzaky Ramadhani, Ahmad Watsiq Maula, Indah Kartika Murni, Emma Watts, Ilma Safira Baehaqi, Rizka Dinari, Danindra Ario Wiryawan, Daniel Daniel, Celeste M. Donato, Julie E. Bines

**Affiliations:** a Department of Biostatistics, Epidemiology, and Population Health, Faculty of Medicine, Public Health, and Nursing, Universitas Gadjah Mada, Yogyakarta, Indonesia; b Center for Child Health - Pediatric Research Office, Faculty of Medicine, Public Health, and Nursing, Universitas Gadjah Mada, Yogyakarta, Indonesia; c Child Health Department, Faculty of Medicine, Public Health, and Nursing, Universitas Gadjah Mada, Yogyakarta, Indonesia; d Enteric Diseases Group, Murdoch Children's Research Institute, Parkville, Victoria, Australia; e Department of Health Behavior, Environment, and Social Medicine, Faculty of Medicine, Public Health, and Nursing, Universitas Gadjah Mada, Yogyakarta, Indonesia; f Department of Paediatrics, The University of Melbourne, Parkville, Victoria, Australia; g Department of Gastroenterology and Clinical Nutrition, Royal Children's Hospital Melbourne, Victoria, Australia

**Keywords:** Typhoid fever, *Salmonella* Typhi, National insurance claim data, Prevalence, Sub-national data

## Abstract

**Background:**

Despite Indonesia's high typhoid burden, subnational prevalence data remain sparse, limiting risk mapping and targeted interventions. We characterised trends in typhoid presentation in primary care and hospital settings, described geographic patterns, and identified factors associated with hospitalisation.

**Methods:**

We conducted a serial cross-sectional study using the multi-year *Badan Penyelenggara Jaminan Sosial (BPJS) Kesehatan* national insurance sample. Typhoid fever cases were identified using ICD-10 codes A01.0-A01.09, with sampling weights applied to generate nationally representative estimates. Temporal trends were assessed using time-series analyses, and factors associated with hospitalisation using Poisson regression.

**Findings:**

From 1 January 2015 to 31 December 2023, an estimated 8,180,668 ICD-10 coded typhoid-related healthcare visits were recorded, including 3,895,695 hospital admissions. Mean annual prevalence peaked at 314 per 100,000 in hospitals (2016) and 347 per 100,000 in primary care (2017), with the highest prevalence among children younger than 5 years. Cases were concentrated on Java. Higher proportion of hospitalisation was observed among BPJS Kesehatan-subsidised participants (aPR 1·07; 95% CI: 1·05–1·09), elderly aged ≥65 years (aPR 1·07; 95% CI: 1·04–1·09), and children (0–5 years: aPR 1·02; 95% CI: 1·01–1·03; 6–18 years: aPR 1·03; 95% CI: 1·02–1·04).

**Interpretation:**

Typhoid fever remains a substantial public health burden in Indonesia despite a declining trend. Higher hospitalisation among subsidised participants and vulnerable age groups reflects persistent socioeconomic and age-related inequities. Control efforts should integrate water and sanitation improvements and vaccination, informed by epidemiological patterns to optimise geographic targeting and dose scheduling.

**Funding:**

Gates Foundation.

**Research in context**

**Evidence before this study**

Typhoid fever remains a major public health threat in Southeast Asia, with Indonesia contributing a considerable share of the regional burden. However, the availability of robust and representative epidemiological data to inform control strategies remains limited. We searched Pubmed from database inception to 31 March 2026 using the terms ‘typhoid fever’, ‘epidemiology’ (including prevalence and incidence), and ‘Indonesia’. We identified nine relevant articles, showing that existing epidemiological evidence is predominantly limited to hospital-based surveillance and reflects a more severe spectrum of disease. One study, based on the Global Burden of Disease (GBD) 2023 estimates, Indonesia has the highest incidence (99 per 100,000 population) and mortality rate (1·29 per 100,000 population) of typhoid fever in Southeast Asia, making it one of the major contributors to the regional and global burden of disease. However, these national-level estimates lack the granularity required to assess subnational variation in disease distribution. In Indonesia, the reliance on clinical diagnosis over microbiological culture due to cost and diagnostic limitations further obscures the true community-level burden. While the emergence of multidrug-resistant *Salmonella* Typhi necessitates urgent preventive strategies such as the Typhoid Conjugate Vaccine (TCV), there is a significant knowledge gap regarding population-based epidemiological trends and geographic heterogeneity across primary care and hospital settings.

**Added value of this study**

This study utilises a large-scale national administrative database to characterise typhoid fever trends in Indonesia over a nine-year period (2015–2023), capturing approximately 8 million healthcare visits, of which 47·62% resulted in hospital admissions. We found that the highest annual prevalence reached 347 per 100,000 in primary care and 314 per 100,000 in hospitals, figures that consistently exceed high-burden thresholds. The study identifies significant subnational geographic clustering on Java Island and highlights specific vulnerabilities through adjusted prevalence ratios (aPR), showing higher hospitalisation among lower socioeconomic groups, the elderly aged ≥65 years (aPR 1·07; 95% CI: 1·04–1·09), and children (0–5 years: aPR 1·02; 95% CI: 1·01–1·03; 6–18 years: aPR 1·03; 95% CI: 1·02–1·04).

**Implications of all the available evidence**

The persistent burden of typhoid fever in Indonesia underscores the need for a multisectoral control strategy. These findings support consideration of integrated interventions, including the introduction of TCV alongside targeted improvements in water and sanitation. The observed subnational inequities suggest that public health impact could be optimised by prioritising high-burden regions and vulnerable populations. Future studies are warranted to strengthen surveillance through complementary approaches, including serosurveillance and emerging innovations such as wastewater surveillance, to address limitations of clinical surveillance, better characterise population-level burden, and evaluate the effectiveness of public health interventions.

## Introduction

Typhoid fever, caused by *Salmonella enterica* serovar Typhi, continues to cause a substantial burden of morbidity and mortality globally, with a disproportionate impact on low- and middle-income countries. Despite its wide clinical spectrum, ranging from mild febrile illness to life-threatening complications, typhoid fever has become an increasing global concern due to the emergence and spread of multidrug-resistant *Salmonella* Typhi which undermine treatment effectiveness and threaten current control strategies.^1^ The Global Burden of Disease (GBD) 2023 study suggests that, although the global incidence of typhoid fever has declined since 1990, the disease remains a major public health problem in South Asia, Southeast Asia, and Oceania. Within Southeast Asia, Indonesia has the highest estimated incidence (99 per 100,000 population) and mortality (1·29 per 100,000 population), making it a major contributor to the regional and global burden.^2^ However, as highlighted in published GBD analyses, estimates do not provide granular subnational data to characterise provincial-level variations in disease distribution, critical for targeted public health interventions.

Estimating the burden of typhoid fever remains a global challenge because of limitations in current diagnostic methods. In endemic settings, the Widal agglutination test has poor sensitivity and specificity due to high background seroprevalence,^3^ and currently available rapid diagnostic tests offer only modest improvements.^4^ Although blood culture is the reference standard, the sensitivity is suboptimal, particularly following prior antibiotic use, and routine use is constrained by laboratory capacity and cost. Consequently, microbiological confirmation is rarely undertaken in routine practice in many low- and middle-income countries, including Indonesia, where diagnosis is largely clinical. Current estimates of incidence and mortality therefore rely mainly on hospital-based surveillance, which preferentially captures severe disease, while routine primary care surveillance, where most uncomplicated cases are managed, remains limited. As a result, culture-based surveillance is likely to underestimate disease burden, whereas clinically based surveillance may overestimate it through diagnostic misclassification. Despite these uncertainties, decisions on preventive interventions, including improvements in water, sanitation, and hygiene and the introduction of typhoid conjugate vaccine (TCV) into national immunisation programmes,^5^ cannot await perfect surveillance systems. Instead, policy should be guided by the best available evidence while acknowledging the limitations of existing data sources.

Indonesia's National Health Insurance (*Badan Penyelenggara Jaminan Sosial Kesehatan*, BPJS Kesehatan) capitation claims sample dataset captures typhoid fever coded using the 10th revision of the International Classification of Diseases (ICD-10) across both primary care and hospital settings and covers approximately 1% of the Indonesian population (N = 2,499,681).^6^ This large-scale administrative dataset enables population- and hospital-level assessment of typhoid fever across diverse care settings and geographic regions not captured by traditional hospital-based surveillance, while allowing timely and cost-efficient estimation of disease burden in settings with limited laboratory confirmation. The BPJS Kesehatan longitudinal sample dataset has considerable potential for health policy analysis by generating nationally representative evidence to monitor disease burden, evaluate healthcare delivery, and inform priority setting and policy decisions.^6^ Leveraging the longitudinal nature of these data, we aim to characterise temporal trends in primary care consultations and hospital admissions, describe geographic and demographic heterogeneity, and identify factors associated with typhoid-related hospitalisation to inform typhoid control policy.

## Methods

### Study design

We conducted a serial cross-sectional study using the BPJS Kesehatan sample dataset between 1 January 2015 and 31 December 2023, which comprised an unweighted sample of 2,499,681 unique individual records. Sampling weights provided by BPJS Kesehatan were applied to generate nationally representative population estimates, capturing healthcare utilisation across both primary care and hospital settings. As of 2023, BPJS Kesehatan reported a total of 267 million registered members, covering approximately 95·9% of the Indonesian population.^7^

### Data source

The BPJS Kesehatan sample dataset comprises anonymised individual-level enrolment data and longitudinal healthcare encounter records, including demographic (age and sex), BPJS Kesehatan membership characteristics (class of care and participant segment), and diagnoses coded using the ICD-10 to ensure consistent disease identification nationwide. Although primarily designed for administrative and reimbursement purposes, its breadth makes it a valuable resource for public health surveillance. In this study, we used capitation data, capturing participants registered at healthcare facilities for whom providers receive fixed monthly payments, irrespective of healthcare utilisation.

A stratified random sample of households was drawn from BPJS Kesehatan membership database to generate nationally, provincially, and district-representative estimates. Households were the primary sampling unit because BPJS Kesehatan enrolment is household-based and healthcare utilisation is likely to be correlated among household members. The sampling design incorporates multi-cohort sub-populations, including historical members and new annual enrolments with sampling strata defined by cross-classifying households according to their registered primary care facility, household healthcare utilisation status, region, and membership type. Within each stratum, households were selected using simple random sampling based on BPJS Kesehatan sampling protocols.^8^

Sampled households were identified within the BPJS Kesehatan membership database, which also provided the corresponding household member records. Healthcare utilisation records from the primary and referral healthcare claims databases were subsequently extracted for each household member. To account for unequal selection probabilities across strata and cohorts, BPJS Kesehatan calculated sampling weights as the inverse of each household's probability of selection.^8^ These weights were applied in all analyses to generate population-representative estimates.

For analyses comparing urban and rural populations, participants were classified according to the district administrative location recorded in the BPJS Kesehatan membership database. As the dataset lacks village-level (desa/kelurahan) identifiers, districts were classified as urban (kota/city) or rural (kabupaten/regency), consistent with Indonesia's official administrative classification. This approach was used as the most granular feasible proxy for residence, as city formation legally incorporates population density and non-agricultural economic activity thresholds,^9^ dimensions also used by Statistics Indonesia to classify urban villages,^10^ and is consistent with the Indonesian rural-definitions literature.^11^ Annual and geographically specific prevalence were calculated using total BPJS Kesehatan memberships obtained from the Indonesian Health Profile (Profil Kesehatan Indonesia) for year 2020–2023^7^^,^^12^, ^13^, ^14^ and the JKN Statistical Book (*Buku Statistik JKN*) for year 2015–2019.^15^ Population density and the proportion of households with access to improved sanitation, as an indicator of household sanitation conditions, were obtained from the Indonesian Health Profile year 2015–2023.^7^^,^^12^, ^13^, ^14^^,^^16^, ^17^, ^18^, ^19^, ^20^

### Case definitions

Typhoid fever cases were identified using physician-assigned ICD-10 codes recorded as either primary or secondary diagnoses, specifically: A01.0 (Typhoid fever), A01.00 (Typhoid fever, unspecified), and A01.01 (Typhoid meningitis). Duplicate records, identified using unique visit identifiers (FKP02 for primary care and FKL02 for referral care), were excluded. Diagnoses were assigned according to the Indonesian Ministry of Health Decree No. HK.01.07/MENKES/1186/2022, which differ by level of care. In primary care, where reimbursement is capitation-based, laboratory confirmation is not routinely required. In hospital settings, supporting laboratory investigations (e.g., Tubex, Typhidot, or, in earlier years, Widal testing) are generally undertaken as part of the diagnostic work-up for reimbursement claims.^21^ Although these investigations do not replace microbiological confirmation, the standardised diagnostic framework provides greater diagnostic consistency than clinical assessment alone.

To avoid overestimating prevalence due to follow-up visits or repeat consultations for the same illness episode, an episode-based filtering approach was applied. Healthcare encounters for typhoid fever occurring within 30 days of a preceding record for the same individual (coded as PSTV01) were considered part of a single clinical episode, and only the final chronological record within each episode was retained for analysis. The 30-day interval was selected based on the natural history of typhoid fever, in which the incubation period typically ranges from 6 to 30 days and the clinical illness generally lasts 3–7 days, whereas relapse most commonly occurs 2–3 weeks after completion of treatment.^22^ As a result, repeated healthcare encounters within 30 days are more likely to represent ongoing treatment or follow-up care rather than a new infection. This interval provides a pragmatic boundary for distinguishing a single illness episode from a subsequent relapse in routinely collected administrative data, although it may merge some true relapses with the index episode and, less commonly, classify prolonged or complicated illness as separate episodes. Results were reported as typhoid fever episodes, with a denominator defined as the regional total BPJS Kesehatan memberships for prevalence.

### Statistical analysis

All statistical analyses were performed using Stata 17 and Python 3.11.9 utilising pandas, statsmodels, geopandas, and matplotlib packages. De-identified data were analysed using time-series analyses to assess temporal trends in both primary care and hospital settings, including age-specific prevalence. To account for annual expansions in insurance coverage, annual prevalence was estimated using the verified BPJS Kesehatan membership in each respective year as the dynamic denominator. In particular, geographic distribution was presented both by administrative provinces and by Indonesian Case Based Groups (INA-CBGs) regionals, which were grouped into five regions, incorporating indicators of consumer price index.

To provide geographic context for the observed typhoid distribution, additional descriptive choropleth maps were generated for healthcare utilisation, households with access to improved sanitation facilities, and population density at the provincial level. Primary care and hospital utilisation rates were calculated from the BPJS Kesehatan sample dataset as annual healthcare visits per 100,000 population, using the mean annual BPJS Kesehatan membership of each province over the study period as the denominator. Data on households with access to improved sanitation facilities and population density were obtained from the Indonesian Health Profile.^7^^,^^12^, ^13^, ^14^^,^^16^, ^17^, ^18^, ^19^, ^20^

Analyses of factors associated with hospitalisation were restricted to typhoid cases treated in hospitals. All descriptive statistics and time-series analyses were weighted to generate population-representative estimates. Weighted counts were rounded to the nearest integer for reporting. Consequently, minor discrepancies may exist between the sums of sub-categories and their respective totals. Adjusted prevalence ratios (aPRs) and 95% confidence intervals (CIs) were estimated using Poisson regression with robust (Huber-White sandwich) variance estimation. This approach is to calculate prevalence ratios for factors associated with typhoid hospitalisation, which provides stable estimates of prevalence ratios for common binary outcomes and avoids the overestimation associated with odds ratios from logistic regression.

### Ethics

The study was approved by the Medical and Health Research Ethics Committee, Universitas Gadjah Mada (KE/FK/1741/EC/2025). The use of anonymised secondary data necessitated a waiver of informed consent per local and institutional guidelines.

## Results

After applying sampling weights to the 2,499,681 unique individuals, the weighted sample represents a national population of approximately 290,450,909 individuals, closely approximating the Indonesian population. Annual coverage of BPJS Kesehatan membership is presented in Fig. 1, which reached up to 95·9% of the Indonesian population in 2023. Flow of data selection for this study is depicted in Fig. 2, including cases presenting to primary care facilities, hospitals, and referral services.

**Fig. 1:**
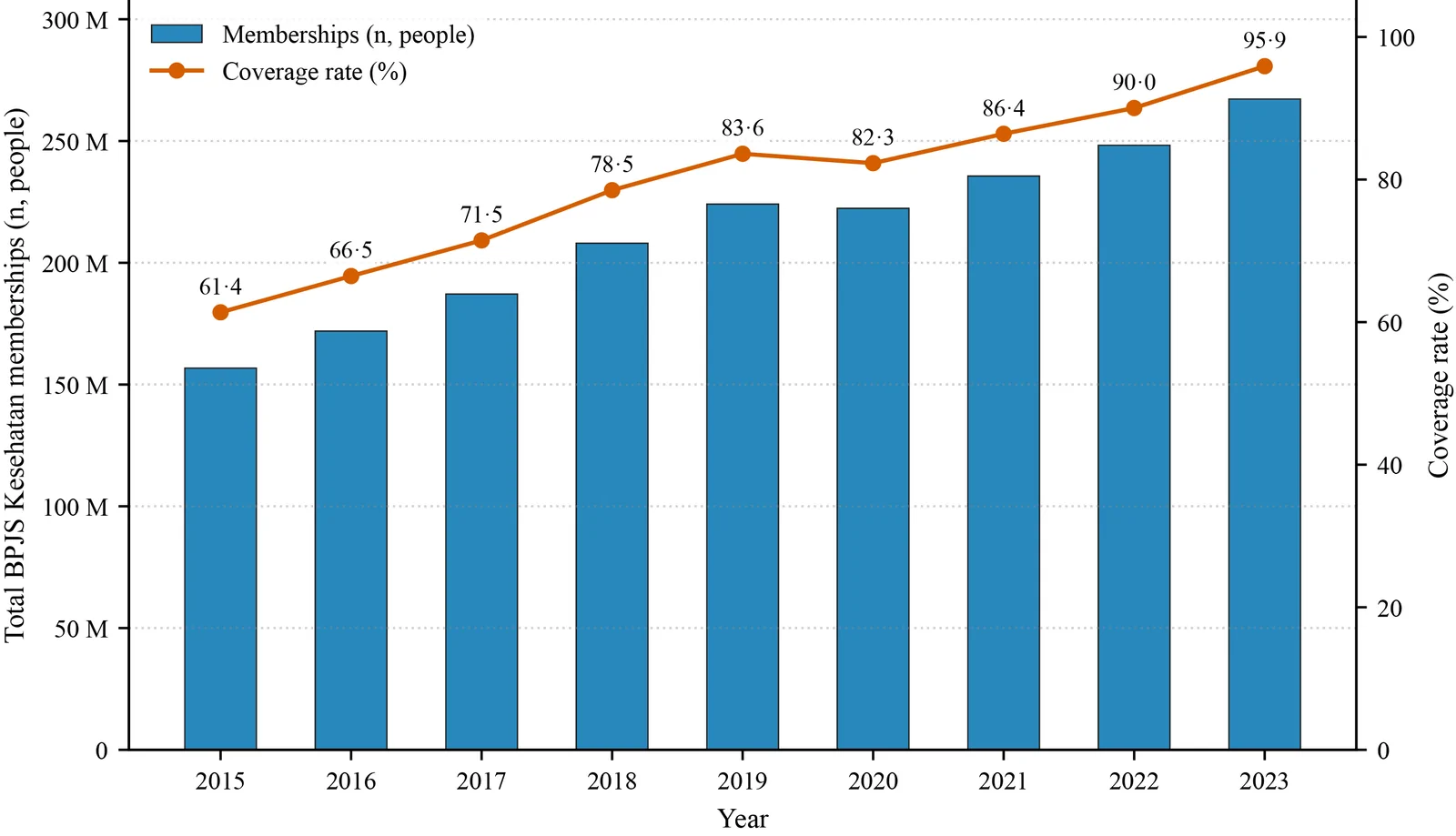
**Annual BPJS Kesehatan membership and coverage rate, 2015–2023**.

**Fig. 2:**
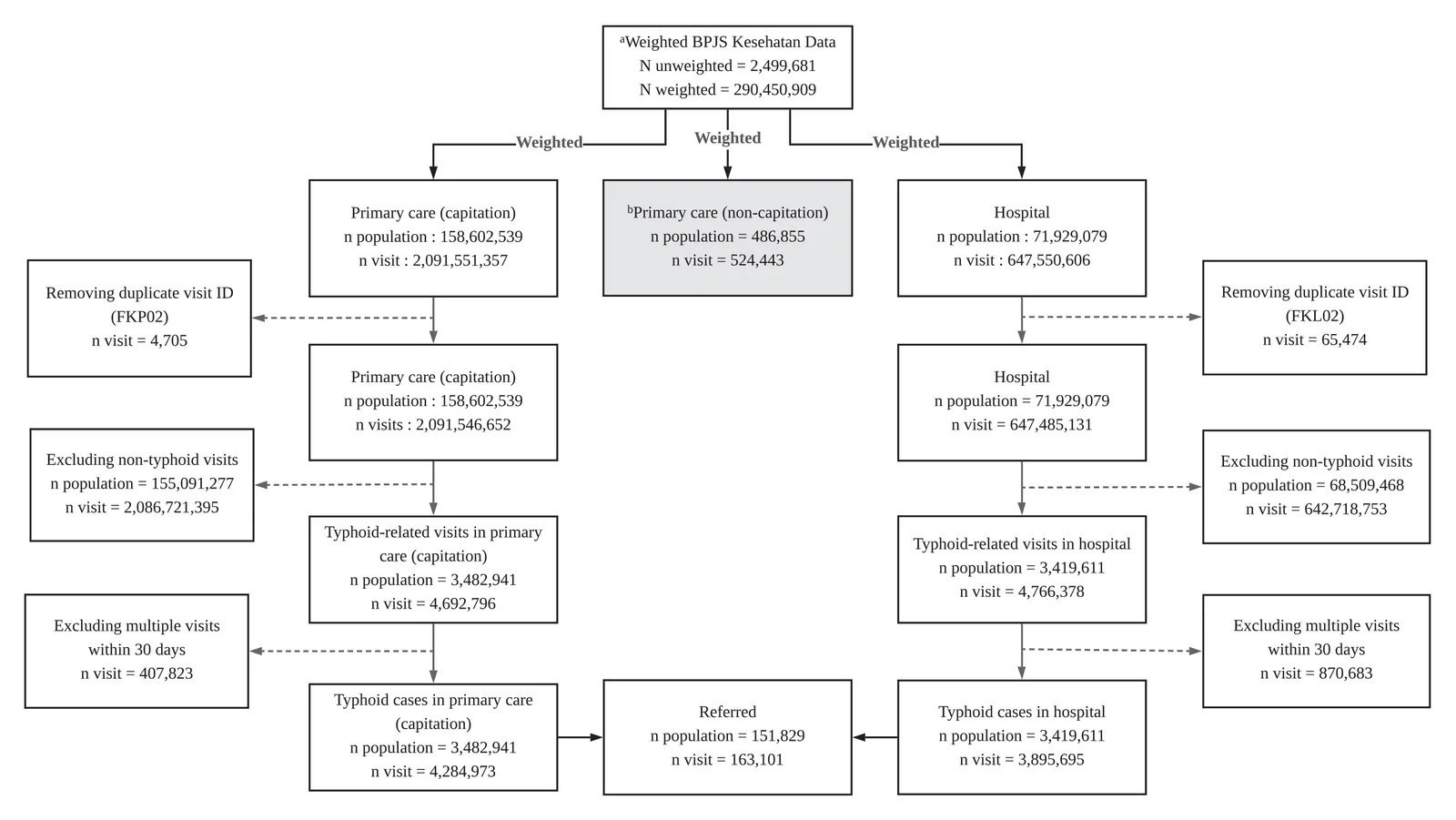
**Structure and flow of BPJS Kesehatan sample data (2015–2023) used in the study.**^a^N population of the unweighted data: 2,499,681, which is 1% of the actual sample before weighting. ^b^Non-capitation data were excluded from this study, as non-capitation represents additional services billed on a fee-for-service basis.

### Sociodemographic characteristics of typhoid cases and visits

Over the nine-year period (2015–2023), 8,180,668 typhoid-related visits were recorded among 6,902,552 individuals, with most visits being diagnosed under ICD-10 code A01.0 (Table 1). No records were identified for ICD-10 codes A01.02-A01.09.

**Table 1:** Total number of weighted visits for typhoid by ICD-10 code.

ICD-10 Code	Description	Primary care	Hospital		Referred^b^	
			Primary diagnosis	Secondary diagnosis^a^	Primary diagnosis	Secondary diagnosis^a^
A01.0	Typhoid fever	4,281,451	3,206,464	690,367	58,042	78,908
A01.00	Typhoid fever, unspecified	2245	0	0	0	0
A01.01	Typhoid meningitis	1277	0	0	0	0

a If the primary diagnosis is already A01.0, then the secondary diagnoses are not counted.

b Participants receiving care in both primary care and hospitals.

Hospital admissions accounted for a substantial proportion of these visits, representing 3,895,695 (47·62% of all visits). Table 2 shows demographic characteristics of typhoid fever from different healthcare facilities. Adults aged 19–65 years constituted approximately half (54·50%) of the total typhoid-related visits, followed by adolescents (29·77%). Across all subgroups, individuals classified as Class III, the most affordable tier with the simplest inpatient ward facilities, contributed the highest proportion of visits. Regarding employment classification, individuals in the formal sector exhibited a higher proportion of healthcare visits compared to those who were subsidised, self-employed, or unemployed.

**Table 2:** Demographic characteristics of weighted healthcare visits diagnosed with typhoid fever (ICD-10 A01.0 and its subgroup), by facility type and referral status from 2015 to 2023.

Variable	Primary care (n = 4,284,973)	Hospital (n = 3,895,695)		Referred^c^ (n = 163,101)	
		Outpatient (n = 822,368)	Inpatient (n = 3,073,326)	Outpatient (n = 148,651)	Inpatient (n = 14,449)
Age group (in years)					
0–5	442,199 (10·32%)	114,085 (13·87%)	428,167 (13·93%)	17,399 (14·11%)	729 (5·03%)
6–18	1,362,199 (31·79%)	218,617 (26·58%)	854,422 (27·80%)	31,770 (25·76%)	3464 (23·87%)
19–64	2,336,469 (54·53%)	467,110 (56·80%)	1,654,956 (53·85%)	64,283 (52·12%)	9132 (62·92%)
≥65	144,104 (3·36%)	22,555 (2·74%)	135,780 (4·42%)	9058 (7·34%)	1109 (7·64%)
Sex					
Male	2,155,632 (50·31%)	399,589 (48·59%)	1,438,772 (46·82%)	62,891 (50·99%)	6895 (47·51%)
Female	2,129,341 (49·69%)	422,779 (51·41%)	1,634,553 (53·19%)	59,621 (48·34%)	7541 (51·95%)
Class of care^a^					
Class I	1,051,919 (24·55%)	320,627 (38·99%)	1,044,933 (34·00%)	41,916 (33·98%)	4614 (31·79%)
Class II	940,755 (21·96%)	209,935 (25·53%)	759,558 (24·72%)	25,118 (20·36%)	2606 (17·96%)
Class III	2,290,384 (53·45%)	290,373 (35·31%)	1,266,223 (41·20%)	55,347 (44·87%)	7216 (49·71%)
Participant segment^b^					
Formal sector	1,903,792 (44·43%)	488,425 (59·39%)	1,588,328 (51·68%)	60,911 (49·38%)	6329 (43·61%)
Subsidised	1,818,803 (42·45%)	174,795 (21·26%)	878,856 (28·60%)	29,227 (23·70%)	5597 (38·56%)
Self-employment	494,875 (11·55%)	143,171 (17·41%)	508,208 (16·54%)	28,194 (22·86%)	2038 (14·04%)
Non-workers	66,203 (1·55%)	15,949 (1·94%)	97,930 (3·19%)	4178 (3·39%)	471 (3·25%)

a Missing data on 6095 visits.

b Missing data on 1323 visits.

c Participants receiving care in both primary care and hospitals.

### Annual trends of typhoid-related visits per total healthcare facility visits

The highest annual prevalence of typhoid-related visits was 347 per 100,000 population in primary care facilities (2017). Hospital prevalence peaked earlier, in 2016, at 314 per 100,000 population, comprising 251 per 100,000 population for inpatient visits and 63 per 100,000 population for outpatient visits. Typhoid case rate decreased markedly in 2020 and 2021, followed by a slight increase in subsequent years. The increase was more pronounced for inpatient than outpatient visits. The most recent annual prevalence in 2023 was 143 and 167 per 100,000 population in primary care and hospital settings, respectively (Fig. 3). Reported cumulative deaths over the 9 year period included 24 from primary care and 11,991 from hospitals, corresponding to average annual mortality rates 0·0013 and 0·57 per 100,000 population, respectively.

**Fig. 3:**
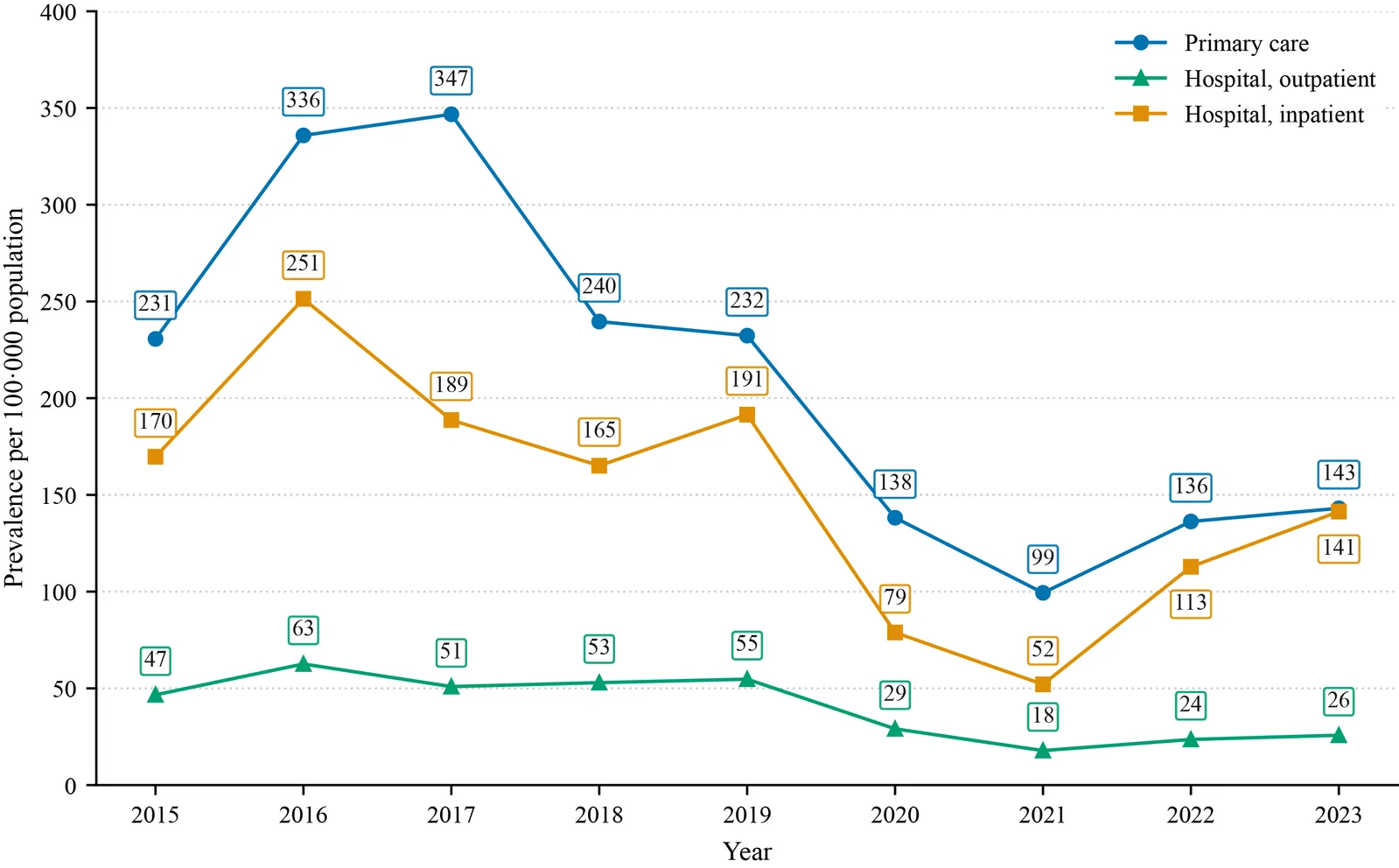
**Prevalence of typhoid fever per 100,000 population (denominator: total annual BPJS Kesehatan membership)**.

### Age-specific prevalence of typhoid-related visits

Although individuals aged 18–65 years accounted for the largest proportion of typhoid-related visits among all typhoid cases (Table 2), prevalence was highest among children aged 0–5 years in both primary care and hospital settings. In contrast, adults aged over 65 years consistently had the lowest prevalence among all age groups across both healthcare settings (Fig. 4).

**Fig. 4:**
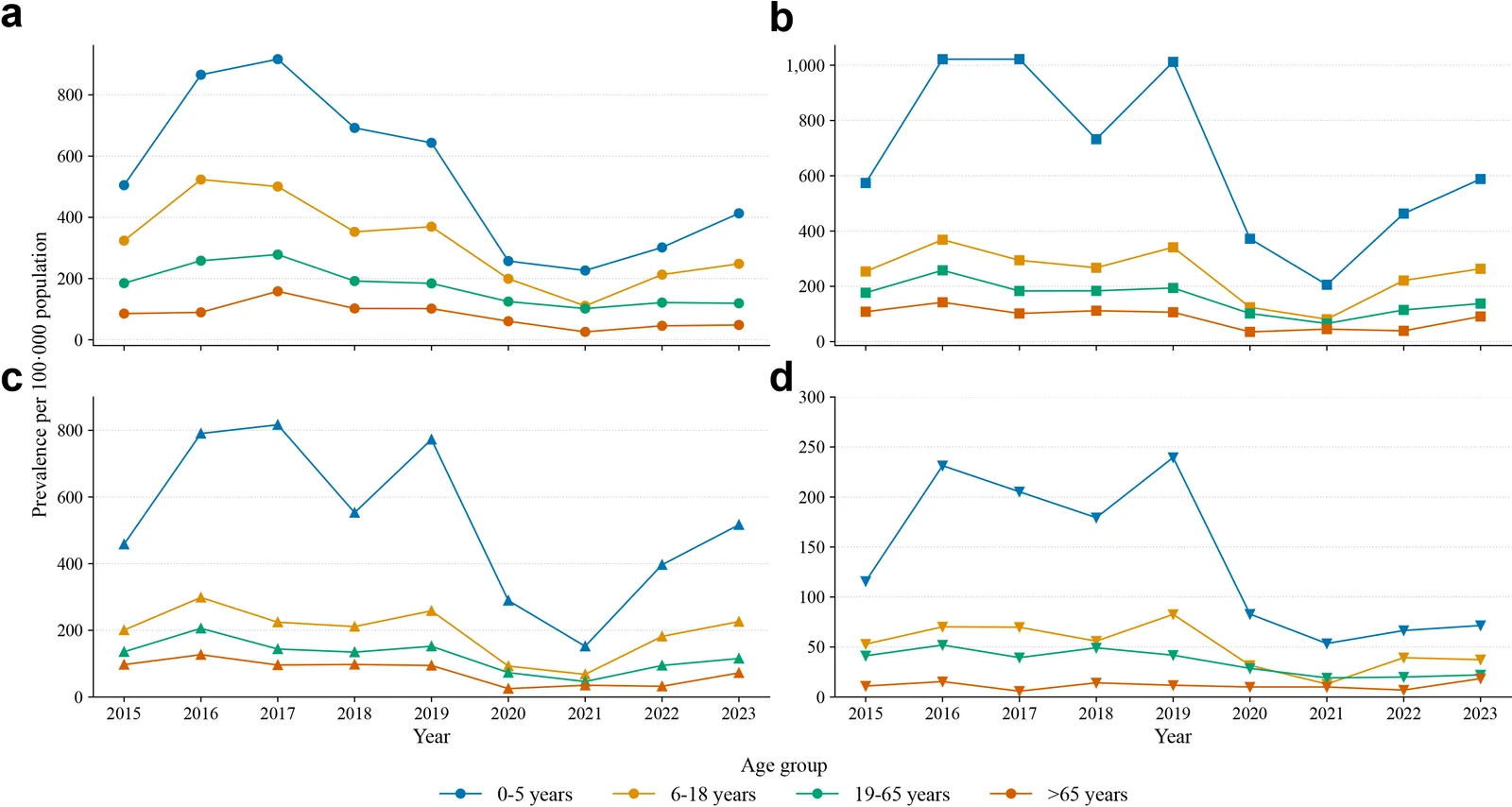
**Prevalence of typhoid fever by age group per 100,000 population (denominator: total annual BPJS Kesehatan membership within the respective age group).** a) Primary care prevalence, b) hospital prevalence, c) inpatient prevalence, and d) outpatient prevalence, with panels c and d providing a breakdown of hospital prevalence shown in panel b.

### Geographical distribution of typhoid cases and healthcare utilisation

Prevalence was consistently higher in urban than in rural areas across the study period in both primary care and hospital settings (Supplementary Figure S1). Geographically, the highest mean annual prevalence in primary care was in Central Java province (302 per 100,000) followed by Bengkulu province (289 per 100,000). In hospital, mean annual prevalence was highest in Aceh province (496 per 100,000 population), followed by Gorontalo province (311 per 100,000) and North Sumatera province (298 per 100,000).

A similar geographic pattern was observed for healthcare utilisation. Primary care utilisation was highest in Java Island, with the highest utilisation observed in the Special Region of Yogyakarta, whereas hospital utilisation was more geographically dispersed. Hospital utilisation was high in Aceh, where typhoid-related hospitalisation was similarly high. In contrast, eastern provinces such as Papua Barat (10 and 11 per 100,000 population in primary care and hospitals, respectively) and Papua (24 and 48 per 100,000 population in primary care and hospitals, respectively) had lower prevalence, consistent with the lower healthcare utilisation observed in these provinces. Outside Java island, provinces with higher percentage of households with access to improved sanitation generally had lower typhoid prevalence, whereas several provinces in Java exhibited both relatively high percentages of households with access to improved sanitation and high typhoid prevalence (Fig. 5).

**Fig. 5:**
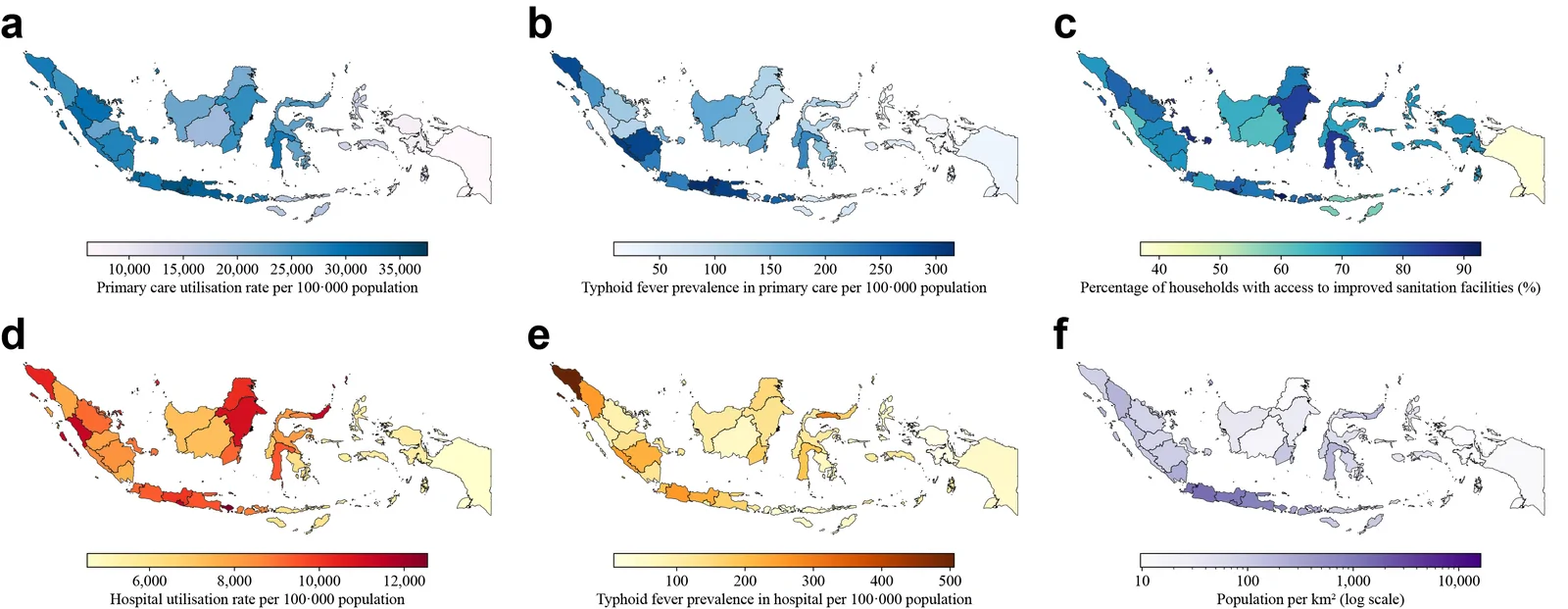
**Geographic distribution of healthcare utilisation, typhoid mean annual prevalence, environmental health, and population density across Indonesian provinces.** a) Mean annual primary care utilisation rate by province, b) Mean annual typhoid prevalence in primary care by province, c) Mean annual percentage of households with access to improved sanitation facilities by province, d) Mean annual hospital utilisation rate by province, e) Mean annual typhoid prevalence in hospitals by province, f) Mean annual population density by province, based on the Indonesia Health Profile. Panels a and d show the rate of BPJS Kesehatan members utilising primary care and hospital services per 100,000 population, respectively. Panels b and e express typhoid prevalence per 100,000 population using the mean annual BPJS Kesehatan membership of each province over the study period as the denominator. Panel c shows the proportion (%) of households with appropriate wastewater management, and panel f shows population density in people/km^2^.

When aggregated based on the INA-CBGs regional classification, the highest annual prevalence was 2367 visits per 100,000 population in region 1 (Java Island, Indonesia's most populous island) for primary care facilities and 2213 visits per 100,000 population in region 3 (covering Sulawesi and parts of Sumatra and Kalimantan) for hospitals (Fig. 6).

**Fig. 6:**
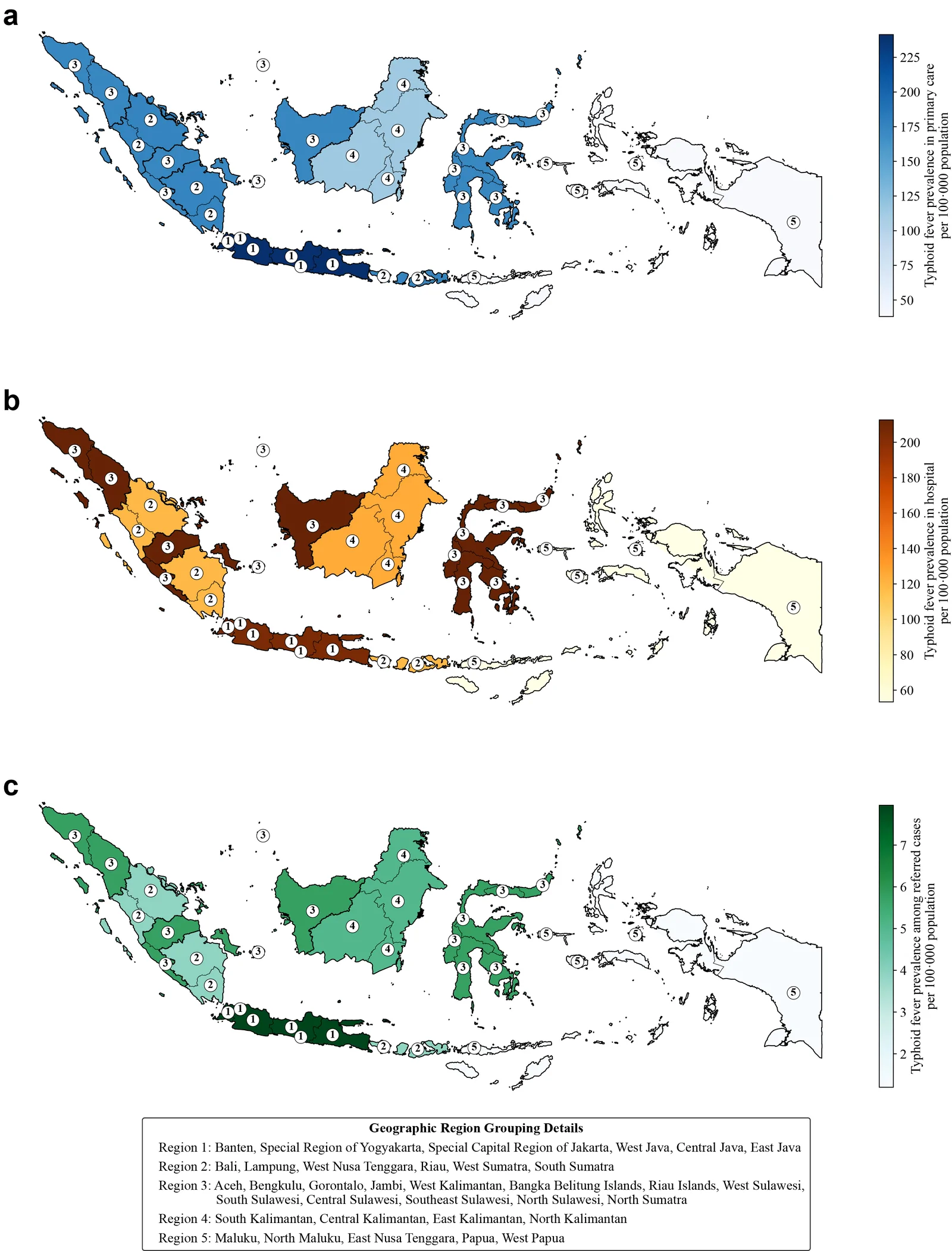
**Mean annual prevalence distribution of typhoid fever by INA-CBGs regional classification per 100,000 population (denominator: mean annual BPJS Kesehatan membership of each region across study years).** a) Mean annual typhoid prevalence in primary care by INA-CBGs regional classification, b) Mean annual typhoid prevalence in hospitals by NA-CBGs regional classification, c) Mean annual typhoid prevalence in referral by NA-CBGs regional classification.

### Determinants of hospitalisation among typhoid patients

Higher hospitalisation was observed among individuals enrolled in the subsidised insurance scheme (aPR 1·07; 95% CI: 1·05–1·09). Elderly aged ≥65 years (aPR 1·07; 95% CI: 1·04–1·09) and children (0–5 years: aPR 1·02; 95% CI: 1·01–1·03; 6–18 years: aPR 1·03; 95% CI: 1·02–1·04) had higher hospitalisation in comparison with adults aged 19–64 years (Table 3).

**Table 3:** Factors associated with hospitalisations among all hospital visits with typhoid fever.^a^

Variable	Outpatient (n = 808,614)	Inpatient (n = 3,016,552)	Prevalence ratio (PR)	95% CI (Lower-Upper)
Region				
Region 5	17,735 (2·19%)	46,631 (1·55%)	ref	ref
Region 1	567,189 (70·14%)	1,828,313 (60·61%)	1·12∗∗	1·08–1·16
Region 2	78,769 (9·74%)	261,028 (8·65%)	1·09∗∗	1·05–1·13
Region 3	119,468 (14·77%)	792,713 (26·28%)	1·21∗∗	1·17–1·26
Region 4	25,451 (3·15%)	87,865 (2·91%)	1·10∗∗	1·05–1·15
Insurance type (FKL12)				
Formal sector	482,909 (59·72%)	1,562,199 (51·79%)	ref	ref
Non-workers	15,180 (1·88%)	95,050 (3·15%)	1·04∗	1·01–1·07
Subsidised	167,819 (20·75%)	855,889 (28·37%)	1·07∗∗	1·05–1·09
Self-employment	142,704 (17·65%)	503,414 (16·69%)	1·03∗	1·01–1·04
Sex (PSTV05)				
Female	416,839 (51·55%)	1,606,590 (53·26%)	ref	ref
Male	391,774 (48·45%)	1,409,961 (46·74%)	0·98∗∗	0·97–0·99
Class (PSTV07)				
Class I	317,269 (39·24%)	1,029,715 (34·14%)	ref	ref
Class II	208,230 (25·75%)	747,447 (24·78%)	1·02∗	1·01–1·03
Class III	283,114 (35·01%)	1,239,390 (41·09%)	1·00	0·98–1·02
Age group (in years)				
19–65	463,104 (57·27%)	1,629,425 (54·02%)	ref	ref
0–5	111,228 (13·76%)	420,886 (13·95%)	1·02∗	1·01–1·03
6–18	467,110 (56·80%)	1,654,956 (53·85%)	1·03∗∗	1·02–1·04
>65	22,555 (2·74%)	135,780 (4·42%)	1·07∗∗	1·04–1·09

∗∗p ≤ 0·001, ∗p ≤ 0·01.

a The multivariate analysis included participants with complete data only.

## Discussion

### Typhoid prevalence: temporal trends and geographical distribution

Over nine years of observation (2015–2023), typhoid remained a substantial public health burden in Indonesia, with hospitalisation disproportionately affecting vulnerable populations. Despite declining trends, the estimated burden remained above the threshold used to define high-burden settings (>100 cases per 100,000 person-years). Our estimates were higher than those reported in Fiji, where crude incidence based on culture-confirmed cases from blood, stool, pus, or other sterile-site specimens was 32·1 per 100,000 population in 2014 and 30·4 per 100,000 in 2015.^23^ Our estimates were also higher than the GBD estimate for typhoid burden in Indonesia.^2^ GBD derives incidence via Bayesian meta-regression of available population-based studies, splitting combined enteric fever incidence into typhoid and paratyphoid using aetiological proportion models. As Indonesia lacks nationwide vital registration and standardised culture-confirmed surveillance, the GBD estimate therefore relies substantially on modelled extrapolation rather than direct empirical measurement.^24^ By contrast, Bangladesh reported an adjusted incidence of blood culture-confirmed *Salmonella* Typhi infection of 913 per 100,000 person-years, based on surveillance across selected tertiary hospitals and a laboratory network.^25^

However, typhoid burden estimates across endemic settings are not directly comparable because surveillance and case-ascertainment methods vary substantially. A review of global enteric fever surveillance identified considerable heterogeneity in study design, adjustment for health-care utilisation, and methods used to estimate incidence, limiting comparisons across settings.^26^ Our study captured clinically diagnosed typhoid through ICD-10-coded health insurance claims, enabling estimation of health-care-associated burden across a large population in a setting where blood culture is not routinely performed. Similar administrative approaches have been used in South Korea, where National Health Insurance claims complement the National Notifiable Disease Surveillance System,^27^ and in the Philippines, where ICD-10-coded data have been used to estimate nationwide typhoid burden.^28^ Cross-country differences in reported typhoid burden should therefore be interpreted in the context of both underlying epidemiological variation and differences in surveillance and case ascertainment.

Although the overall trend was downward, some fluctuations were observed during specific periods, with noticeable differences between trends in primary care and hospital. The divergence between primary care and hospital trends during 2016–2017 (Fig. 3) could potentially be related to health system changes rather than typhoid epidemiology. During the early phase of BPJS Kesehatan implementation, rapid membership expansion, evolving referral policies, and maturation of coding practices may have influenced observed trends.^29^ Furthermore, a marked decline was observed during the COVID-19 pandemic, likely reflecting shifts in health-service prioritisation towards COVID-19 care, and reduced health-care utilisation, consistent with the overall decline in health-service use during this period (Supplementary Figure S1). Studies from Taiwan and from Pakistan reported reductions in typhoid cases during the pandemic, attributed in part to strict border controls that may have reduced importation from endemic countries.^30^^,^^31^ Additional explanations include the implementation of non-pharmaceutical interventions, such as social distancing and improved hand hygiene, which may have limited transmission. Following the declaration of the end of the pandemic, case numbers increased but remained below pre-pandemic levels, consistent with global trends indicating a gradual decline in typhoid burden.^32^ The increase in typhoid cases observed in 2022, which was more pronounced among inpatients than outpatients, may reflect the restoration of routine inpatient services as COVID-19 admissions decreased, rather than a true increase in disease severity. This is likely because, during the COVID-19 pandemic, hospitals were required to reallocate approximately 20–40% of general ward beds for COVID-19 care, while shortages of medical supplies, increased demand for limited healthcare resources, and reduced availability of routine health services substantially disrupted healthcare utilisation.^33^

Substantial geographic heterogeneity was observed at the subnational level of cases distribution. The majority of outpatient and inpatient cases were concentrated in provinces classified within INA-CBGs regions 1 and 3, indicating pronounced subnational inequities in disease burden. Disease distribution was highest in densely populated provinces in Java (INA-CBGs region 1), which contribute approximately 56% of the country's population.^34^ Geographic clustering may reflect the combined influence of population density and other contextual factors. One possible explanation is variation in water, sanitation, and hygiene (WASH) conditions. Previous studies have reported widespread use of shallow contaminated groundwater as a household drinking water source, and poor sanitation management in dense and informal urban settlement in Java.^35^^,^^36^ Similarly, our study also found higher cases in parts of Sumatra and Sulawesi (INA-CBGs region 3) where access to adequate WASH infrastructure was mostly below the national figure.^37^ These areas are characterised by rapid urbanisation, peri-urban settlements, and persistent gaps in safe water supply and sanitation coverage. Although typhoid transmission is known to be associated with crowded living conditions, which facilitate faecal-oral spread, particularly in settings where WASH infrastructure is inadequate, our study did not directly measure these factors. The observed spatial patterns may reflect the intersection of demographic pressure, socioeconomic disadvantage, and structural deficiencies in environmental health systems. In this context, the observed geographic patterns provide population-level evidence that may complement other data when prioritising public health interventions, including vaccine implementation and WASH improvements.

### Factors associated with typhoid hospitalisation

Hospitalisation was disproportionately higher among individuals from low-income households, such as those who were BPJS Kesehatan-subsidised patients, reflecting underlying socioeconomic vulnerability and potential barriers to timely access to care.^38^ Individuals at the extremes of age, particularly older adults, had a higher likelihood of hospital admission. In contrast, Garrett et al., using a hybrid surveillance approach that combined facility-based blood culture confirmation with community healthcare utilisation surveys in Bangladesh, Nepal, and Pakistan, did not report a higher incidence among older adults. This may be attributable to their age categorisation, in which individuals aged 25 years and older were grouped into a single category, limiting age-specific interpretation, and precluding direct comparison with our more granular age-stratified estimates.^25^ Notably, although adults of productive age (18–65 years) accounted for the largest proportion of the cases, consistent with Garrett et al., we observed the highest prevalence of typhoid fever among children under 5 years. This finding further supports the importance of early TCV vaccination, particularly from 6 months of age, when children enter a period of increased vulnerability to typhoid fever. WHO evidence indicates that a single dose of TCV administered to children aged 6–11 months or 12–23 months elicits high anti-Vi antibody titres, which persisted for up to 5 years in approximately 84% of children.^39^

Similarly, estimates from the GBD 2017 study identified older and younger age, and low socioeconomic status as important determinants of severe typhoid outcomes, underscoring the vulnerability of populations at the margins of the age spectrum.^40^ These patterns are biologically plausible and may reflect increased susceptibility to infection, greater risk of complications, and more cautious clinical management in these age groups, or a lower threshold for hospital admission. Although adults aged over 65 years had the lowest prevalence of typhoid-related visits, they had a higher likelihood of hospital admission. This may reflect differences in healthcare utilisation and clinical decision-making, as older adults are more likely to have comorbidities, frailty, and a lower threshold for hospital admission, rather than a higher prevalence or greater severity of typhoid fever. Although TCV is not currently routinely recommended for adults aged over 65 years, evidence from a clinical study suggests that TCV is well tolerated in older adults and elicits antibody responses comparable to those observed in younger adults.^41^ Collectively, these findings underscore the intersection of age-related vulnerability and social disadvantage in shaping severe disease trajectories and healthcare utilisation in typhoid fever.

### Why is a high burden of typhoid important?

A persistently high burden of typhoid fever has substantial clinical, epidemiological, and economic implications. Beyond its contribution to morbidity and hospitalisation, sustained transmission reflects ongoing structural inequities in access to safe water, sanitation, and timely healthcare. In endemic settings, a high incidence not only signals continued environmental exposure but also amplifies the cumulative demand for antimicrobial treatment, thereby accelerating the emergence and spread of drug resistance. As such, understanding and addressing typhoid burden is critical not merely for disease control, but for safeguarding the effectiveness of existing diagnostics, therapeutics and strengthening health-system resilience. There have been increasing reports of antimicrobial resistance associated with *Salmonella* Typhi globally, posing a growing challenge to effective clinical management. The emergence and spread of multidrug-resistant and extensively drug-resistant strains have been documented across several endemic settings. Indonesia has been identified as one of the countries reporting reduced susceptibility and resistance to commonly used first-line antibiotics,^42^^,^^43^ underscoring the need for strengthened antimicrobial stewardship, enhanced laboratory surveillance, and preventive strategies, including vaccination and improvements in WASH. Furthermore, imported typhoid cases linked to travel from Indonesia have been reported in a study from Taiwan,^30^ supporting evidence that Indonesia remains an endemic setting and a potential source of international case exportation.

In 2018, following a 2017 WHO's Strategic Advisory Group of Experts (SAGE) review, WHO recommended that typhoid-endemic countries introduce a single-dose TCV for children aged ≥6 months, with catch-up vaccination up to 15 years where feasible, based on demonstrated safety and efficacy and rising antimicrobial resistance.^44^ As of 2025, several Asian countries had progressed towards TCV introduction, with Nepal and Pakistan having incorporated TCV into their routine immunisation programmes and Bangladesh introducing it in 2025,^45^ while India and Myanmar continued to advance preparatory and policy processes for introduction. Indonesia, however, continues to face barriers related to financing constraints and competing public health priorities.^46^ Ongoing policy discussions have highlighted the perceived lack of robust, contemporary epidemiological data to characterise the current disease burden, an evidence gap that our study seeks to address. In parallel, our environmental surveillance findings demonstrate *Salmonella* Typhi positivity in 13% of 406 wastewater samples collected from treatment plants, manholes, river sites, and public spaces, suggesting persistent community-level transmission.^47^ Taken together, our population-based burden estimates and complementary environmental surveillance data provide converging evidence of sustained typhoid transmission in Indonesia, strengthening the case for informed policy deliberation on TCV introduction and broader preventive strategies.

The clinical implications of this study are substantial. Given the persistently high burden of disease, there is a need to strengthen access to reliable diagnostic standards to ensure accurate case identification and appropriate management. Underdiagnosis or misdiagnosis may contribute to ongoing transmission and the persistence of chronic carriers, thereby sustaining community-level spread. From a public health perspective, the substantial burden of typhoid underscores the importance of preventive strategies, including the introduction or expansion of typhoid conjugate vaccination and sustained improvements in WASH infrastructure. The subnational heterogeneity identified in this study provides an opportunity to implement geographically targeted interventions in high-burden areas, thereby maximising impact, and resource efficiency. Indonesia currently lacks comprehensive laboratory-based surveillance to accurately characterise typhoid fever in this endemic setting. The existing Early Warning Alert and Response System *(Sistem Kewaspadaan Dini dan Respons*, SKDR) relies on clinically suspected cases without routine laboratory confirmation. Strengthening surveillance through complementary approaches, including serosurveillance and emerging innovations such as wastewater surveillance, is needed to better estimate population-level burden, inform vaccine policy and scheduling, and evaluate the effectiveness of public health interventions.

### Strengths and limitations

This study has several strengths. It draws on nationwide, multi-year BPJS Kesehatan data covering all Indonesian provinces and both primary care and hospital settings, enabling assessment of temporal trends, hospitalisation patterns, and subnational variation. The large sample provides substantial statistical power, and the demographic distribution broadly reflected the national population reported by Statistics Indonesia, supporting the generalisability of our findings.^48^

Several limitations warrant consideration. Although BPJS Kesehatan covers nearly the entire Indonesian population, this study relied on healthcare utilisation data rather than active population-based surveillance. Typhoid burden may therefore have been underestimated in populations with lower healthcare utilisation, particularly in rural and underserved areas, and geographic differences in healthcare access and care-seeking behaviour may have contributed to the observed regional variation. Similarly, lower hospital utilisation among subsidised participants suggests socioeconomic differences in access to hospital-based care, although BPJS Kesehatan membership class of care is an imperfect proxy for socioeconomic status (Supplementary Figure S2). With the exception of subsidised participants, who are all enrolled in Class III, members of other insurance types, such as self-employed and non-worker participants (excluding government pensioners), can select their preferred ward class (from Class I to Class III). Therefore, insurance type and class do not always reflect socioeconomic status. In addition, disruptions to healthcare utilisation during the COVID-19 pandemic may have influenced temporal trends independently of changes in typhoid epidemiology.

Case ascertainment relied on physician-assigned ICD-10 codes without linked clinical or microbiological confirmation, and subclinical infections and chronic carriers were unlikely to be captured. Despite adherence to national clinical and reimbursement guidelines, overlap with other endemic febrile illnesses, including dengue, leptospirosis, malaria, and rickettsial infections such as scrub typhus, might have resulted in diagnostic misclassification. Supporting laboratory investigations are generally undertaken in hospitals, and over 70% of hospitalised patients in this study had a length of stay exceeding 3 days, consistent with clinically significant bacterial illness. Estimates might therefore be overestimated through misclassification of other febrile illnesses as typhoid fever, or underestimated through missed cases among individuals who did not access BPJS Kesehatan-covered healthcare, were self-treated, or received alternative diagnoses. The overall direction of bias cannot be determined, and any resulting misclassification is likely to be non-differential, tending to attenuate rather than inflate observed associations.

Beyond these ascertainment limitations, the high proportion of hospitalisation should not be interpreted as a direct measure of disease severity, but rather as reflecting health-care utilisation associated with typhoid. Admission is influenced by clinical status, referral and admission practices, access to care, and reimbursement mechanisms, while BPJS Kesehatan data lack clinical, laboratory, and validated severity measures. Furthermore, visits preceding the first typhoid-coded diagnosis were not captured, which might have influenced the observed proportion of hospitalised cases and should be considered when interpreting our findings.

As this study used administrative claims data, diagnostic coding might have been influenced by documentation quality, coding practices, and reimbursement processes, and could not be validated against clinical or laboratory records. Our estimates should therefore be interpreted as the burden of physician-coded typhoid requiring health-care utilisation rather than microbiologically confirmed disease. We identified rare, clinically implausible coding patterns, including A01.01 (typhoid meningitis) recorded exclusively in primary care, likely reflecting coding or administrative inconsistencies. These anomalies represented a very small proportion of the dataset, were confined to a few facilities and districts, and occurred predominantly during early BPJS Kesehatan implementation, consistent with coding maturation and system adjustment. Any resulting bias is therefore more likely to affect absolute estimates than the overall epidemiological patterns.

### Conclusion

Despite declining prevalence, typhoid fever remains a substantial public health burden in Indonesia, with pronounced subnational inequities and disproportionate impact on socioeconomically vulnerable populations and individuals at the extremes of age. The concentration of cases in densely populated areas with inadequate WASH infrastructure underscores persistent structural drivers of transmission. Strengthening diagnostics, antimicrobial stewardship, typhoid conjugate vaccination, and targeted WASH improvements is critical to reduce burden and inequities. In Indonesia's high-morbidity, low-mortality context, intervention strategies should prioritise integrated approaches, with vaccination policies informed by epidemiological evidence to optimise targeting, scheduling, and booster use. Ongoing surveillance, strengthened through integration with innovative approaches such as serosurveillance and wastewater surveillance, is essential to inform and guide public health actions and interventions.

## Contributors

*Conceptualisation*: VO, AWM, EW, ISB, CMD, IKM, JEB.

*Methodology*: VO, ADR, AWM, EW, ISB, CMD, IKM, JEB.

*Validation*: VO, AWM, EW, DD, CMD, IKM, JEB.

*Formal analysis*: VO, ADR, AWM.

*Investigation*: VO, AWM, ISB.

*Data curation*: VO, ADR, AWM, ISB, RD, DAW, IKM.

*Writing–original draft*: VO.

*Writing–review and editing*: VO, ADR, AWM, EW, ISB, RD, DAW, DD, CMD, IKM, JEB.

*Visualisation*: VO, ADR, AWM.

*Supervision*: VO, EW, CMD, IKM.

*Project administration*: VO, ISB, RD.

VO and ADR accessed and verified the data. All authors had full access to all the data in the study and accepted the responsibility to submit it for publication. All authors have read and approved the final manuscript.

## Data sharing statement

All data used in this study are available and can be accessed via the BPJS Kesehatan website (https://data.bpjs-kesehatan.go.id) upon request.

## Editor note

The Lancet Group takes a neutral position with respect to territorial claims in published maps and institutional affiliations.

## Declaration of interests

The authors have no competing interests to declare.
