# Supplementary material for: Burden of typhoid fever in primary and hospital care in Indonesia: a serial cross-sectional study using national health insurance (BPJS Kesehatan) data, 2015–2023
Source: Lancet Reg Health West Pac. 2026 Oct 1;75:101994. doi: 10.1016/j.lanwpc.2026.101994 (PMC13659677; doi:10.1016/j.lanwpc.2026.101994)
Supplement: Supplementary file 1 [file mmc1.docx]

Supplementary Data

Burden of typhoid fever in primary and hospital care in Indonesia: a serial cross-sectional study using national health insurance (BPJS Kesehatan) data, 2015-2023

**Appendix**

**Table of contents**

A. Healthcare utilisation by urban and rural residence	2

B. Healthcare utilisation by socioeconomic	3

# A. Healthcare utilisation by urban and rural residence

To further describe patterns of healthcare utilisation across different residential settings, **Figure S1** presents healthcare utilisation rates per 100,000 population according to urban and rural residence. The figure shows utilisation rates for primary care and hospital healthcare settings, allowing comparison of healthcare-seeking patterns between urban and rural populations.

**Figure S1. Healthcare utilisation rate per 100,000 population by urban and rural residence**. a) Primary care utilisation rate, b) Healthcare utilisation rate.

# B. Healthcare utilisation by socioeconomic

To further characterise differences in healthcare utilisation across socioeconomic groups, **Figure 3** presents healthcare utilisation rates per 100,000 population according to BPJS class and insurance type membership. The figure compares utilisation patterns across BPJS classes and insurance types in both primary care and hospital settings.

**Figure 3. Proportion of healthcare utilisation cases per 100,000 population in primary care and hospital, by BPJS class and insurance type membership.** a) Primary care utilisation rate by BPJS class, b) Primary care utilisation rate by insurance type, c) Hospital utilisation rate by BPJS class, d) Hospital utilisation rate by insurance type.
